# Case Report of Human Urinary Myiasis Caused by *Clogmia albipunctata* (Diptera: Psychodidae) with Morphological Description of Larva and Pupa

**Published:** 2017-12-30

**Authors:** Nadia Ali El-Dib, Wegdan Mohamed Abd El Wahab, Doaa Ahmed Hamdy, Mona Ibrahim Ali

**Affiliations:** 1 Department of Medical Parasitology, Cairo University, El Manial, Cairo, Egypt; 2 Department of Medical Parasitology, Beni-Suef University, Beni-Suef, Egypt

**Keywords:** Urinary myiasis, *Clogmia albipunctata*, Egypt

## Abstract

**Background:**

Urinary myiasis is a form of myiasis caused mainly by larvae of *Fannia scalaris*, *Musca*, *Sarcophaga*, *Lucilia*, *Wohlfahrtia*, *Calliphora*, and rarely by *Eristalis* and *Clogmia albipunctata.*

**Methods:**

This report presents a case of female patient complaining of dysuria and frequency of micturition associated with intermittent passage of small, motile, dark-colored worm-like organisms in urine. She was a married housewife aged 24 years old referred from the Tropical Outpatient Clinic of Beni-Suef University Hospital, Egypt. The patient was subjected to a full questionnaire sheet and investigations such as CBC, stool and urine analysis and urinary ultrasonography. Collected larvae and pupae from urine samples were examined macroscopically and microscopically.

**Results:**

The examined larvae and pupae belonged to *C. albipunctata*. Ivermectin was prescribed to the patient with complaint withdrawal and complete disappearance of the larvae from urine.

**Conclusion:**

This study reports the first case of urinary myiasis caused by *C. albipunctata* in Beni-Suef Governorate, the second in Egypt and third case worldwide. The study throws some light on the medical importance and management of urinary myiasis.

## Introduction

Myiasis is the infestation of human or animal tissues by dipterous fly larvae of the class Insecta. Urogenital myiasis is a rare form of myiasis occurring in humans (1). Like all other types of myiasis, urinary myiasis is usually associated with poor hygienic measures, lack of cleanliness and disability. It may be associated with urinary symptoms as dysuria, hematuria, and obstruction of the urinary system (2).

Urinary myiasis is caused by larvae of *Fannia scalaris*, *Musca*, *Sarcophaga*, *Lucilia*, *Wohlfahrtia,* and *Calliphora.* Few cases of urinary myiasis were caused by *Eristalis* and *Psychoda* flies (3). The family Psychodidae includes six subfamilies, only two of them have medical importance for humans, Phlebotominae (sand flies) which are bloodsuckers and vectors of leishmaniasis and Psychodinae (moth flies) not adapted for blood sucking. *Clogmia albipunctata* is Nematocera of the family Psychodidae, subfamily Psychodinae. Adults live in dark and moist areas including bathrooms and toilets and can be frequently found in drains or sewers hence are called “filter flies or drain flies or bathroom flies”. Larvae live in and feed on decaying organic matter (4).

Myiasis occurs worldwide with more cases being reported from tropical, subtropical and warm temperate areas. In Egypt, some cases of human urogenital myiasis were previously reported (3, 5–8). These detected cases of urinary myiasis in Egypt throw some light on the medical importance and management of this disease in our area.

## Case presentation

A married housewife patient aged 24yr old was referred from the Tropical Outpatient Clinic of Beni-Suef University Hospital, Egypt complaining of intermittent passage of small, motile, dark-colored worm-like organisms in urine. The condition started two years ago associated with dysuria and frequency of micturition. At periods of larval passage, which ranged from two to three days, about 3–4 larvae were seen in each voided urine sample. This was followed by periods of symptoms remission, concomitant with invisibility of the larvae for weeks. However, there was no fever, no hematuria or itching in the periurethral and genital regions.

The patient was living in Ashmant, a rural area in Beni-Suef Governorate, Egypt in poor hygienic conditions. The patient gave history that she urinates in unsanitary toilets “cabinet” or “pit latrine”, with presence of abundant mosquitoes and flies in the bathroom. Therefore, for lack of water pipes, water used for rinsing and bathing was stored in wide uncovered buckets in the bathroom. There was no relationship with farming or livestock rising. No history of traveling or previous operations. Consent was taken from the patient.

Investigations such as CBC, stool analysis “with direct and concentrated smears” and urinary ultrasonography were done and all revealed no abnormalities. The patient was asked to collect urine samples in clean tightly covered containers at periods of larval passages. Complete urine analysis revealed amorphous urate crystals while the numbers of red blood cells (1–3/HPF) and pus cells (5–7/HPF) in urine were within the normal range. Collected larvae and pupae were examined macroscopically and microscopically at the Parasitology Department of Faculty of Medicine, Beni-Suef University, Egypt.

### Macroscopical examination of the larvae and pupae

By naked eye examination, the larva was cylindrical in shape with rounded ventrally curved anterior end and tapering posterior end. The body of the larva was segmented and covered by short seta. Larvae were dark brown to black in color, rapidly motile when freshly passed with different sizes ranging from 8–12mm in length. In addition, identified pupae in freshly passed urine were actively motile, pyriform in shape with cephalothorax carrying two antennae and segmented abdomen. The size of pupa ranged from 4–5mm in length. Four fresh larvae from the patient’s urine were placed in a clean container supported with nutrition in the form of few sugar particles and covered with gauze in a trial for breeding them into adults and were followed up daily. The larvae remained alive for about two weeks but were not able to develop into adult flies. Dead larvae were placed in 5% formalin for fixation.

### Microscopical examination of the larvae and pupae

Larvae fixed in 5% formalin, were washed several times in saline and then incubated in transparent glass bottles containing 30% potassium hydroxide until they became transparent under the microscope. Subsequently, larvae were washed thoroughly in distilled water, dehydrated in ascending grades of ethyl alcohol (30%, 50%, 70%, 90% and 99%) for 30min each, and then in xylene for 30min, mounted in Canada balsam and dried in the oven at 38 °C for two days (9). The mounted larvae were examined microscopically and photographed.

### Description of full-grown larva

**Fig. 1.**
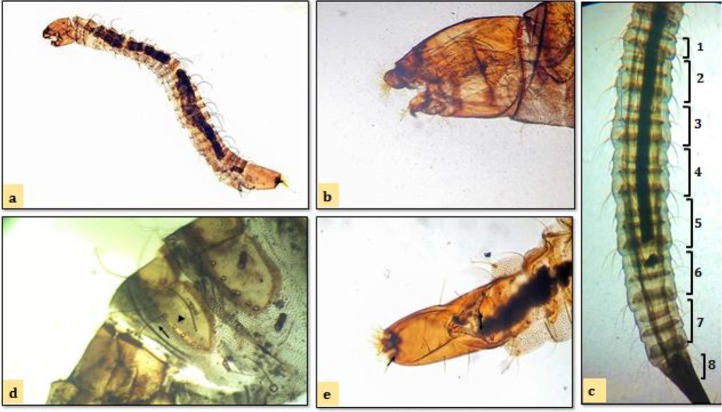
**a)** *Clogmia albipunctata* full larva with elongated, hairy, segmented body (×40). **b)** Anterior part of the larva showing the triangular head (×200). **c**) The dorsal aspect of the abdominal segments showing the saddle-shaped chitinous plates and the subdivisions (×40). **d**) Lateral aspect of the chitinous plates showing long filiform setae (arrow) arising from rounded plates (arrowhead) (×100). **e)** Caudal part showing the ventral aspect of the siphon with spinose anal papilla (double arrow) and the apical posterior spiracles (arrow) (×200)

The microscopically examined larvae were identified as larvae of *C. albipunctata* of different stages depending on the following characters. The larvae were hairy, segmented and yellowish brown in color. The anterior end, posterior end and dorsal plates were slightly darker, while the ventral aspect was slightly lighter in color than the rest of the body. Their length ranged from 8 to 12mm. The body of the larva consisted of the head and eleven segments, 3 thoracic and 8 abdominal segments (Fig. 1a). The head is triangular, not retracted into the thorax with 2 min hairy antennae and 2 ventral mandibles. The mandibles are opposing each other so can be moved against each other on a horizontal plane (Fig. 1b). The dorsal and lateral aspects of the body segments are covered with 26 saddles shaped chitinous plates, 2 for each thoracic segment and 3 for each abdominal segment except the first abdominal segment with two plates and the eighth one without plates. Accordingly, each segment appears divided into several subdivisions (secondary annuli) by these chitinous plates (Fig. 1c). These plates are covered dorsally and laterally with long dark backwardly directed filiform setae arising from rounded chitinous button–shaped basal plates (Fig. 1d). The prothorax has a pair of anterior respiratory spiracles while posterior respiratory spiracles are located on the apical part of the respiratory tube on the siphon. The siphon is cone-shaped, longer than broad (4:1) and its ventral aspect showed spinose anal papillae (Fig. 1e).

### Description of pupa

**Fig. 2.**
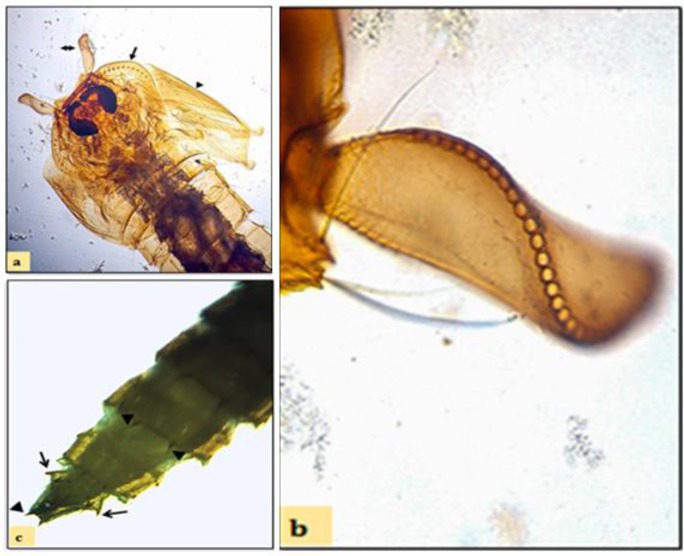
**a)** Anterior part of *Clogmia albipunctata* pupa showing the head with dark eyespots, antenna (arrow), and two respiratory trumpets (double arrow), the thoracic segments with wings of an emerging adult (arrowhead) and the anterior abdominal segments (×40). **b**) Anterior part of the pupa showing the respiratory trumpets with the breathing tubes openings (×400). **c**) Abdominal segments of the pupa showing the lateral processes (arrow) and caudal processes (arrowhead) (×40)

The pupa is pear-shaped and measures 4–5mm in length, with cephalothorax, and 7 visible abdominal segments. The head carried 2 dark eye spots, 2 beaded antennae and 2 funnel-shaped respiratory trumpets in the anterior end. The thoracic segments show the wings of an emerging adult (Fig. 2a). The respiratory trumpet appeared lanceolate in shape including fine circular breathing openings arranged spirally in a rosary shape with few long hairs at the base of the trumpet (Fig. 2b). Each abdominal segment is rectangular in shape with 2 lateral and 2 caudal spine-like processes seen ventrally except the last segment provided only with 2 caudal processes (Fig. 2c).

On the follow-up visits, the patient was advised to take a single oral dose of ivermectin (200ug/kg), to drink plenty of water to help expel any other larvae in the bladder, to keep the water bucket tightly covered in the bathroom and to use spraying insecticides to kill any insects and larvae on the walls and floor of the toilet. She was also advised to change the pit latrine to a toilet seat. After one week, all the patient’s complaints improved with gradual reduction in number and frequency of passing of larvae in urine until complete disappearance of the larvae after treatment. The patient was followed up two months later without recurrence of symptoms or voiding larvae in urine.

## Discussion

This work reports the first case of human urinary myiasis caused by *C. albipunctata* in Beni-Suef Governorate, Egypt. In this case report, the patient had low standard of living in a rural area with poor personal hygiene all considered predisposing factors increasing the risk of urogenital myiasis (10). In addition, the stored water in the uncovered buckets in the bathroom used for rinsing and bathing supported a good medium for insects breeding. The fly eggs are laid in or around the moist urogenital orifices when the patient was urinating, then after hatching; the larvae ascend the urinary tract to feed causing myiasis. This mechanism was previously suggested (7, 11) and is expected to be the cause with our patient.

In spite of the minor medical importance of *C. albipunctata* adults, as they are only nuisance pests, very poor flyers and cannot travel far from their pupation site, their dead bodies may disintegrate to form potential allergens (4). They have been also incriminated as potential mechanical vectors of some bacterial pathogens and were responsible for nosocomial infections (12). *Clogmia albipunctata* larvae were reported as cause of nasopharyngeal myiasis (13), intestinal myiasis (14), and urinary myiasis (15).

Larvae of *C. albipunctata* could be differentiated from other members of genus *Psychoda* (especially *P. latreille* and *P. albipennis*) by having much darker body coloration and the 26 dorsal plates while other members of genus *Psychoda* are lighter in color and the number of dorsal plates is less than 26. Moreover, the siphon of *P. albipennis* is slender, 7 to 8 times as long as broad (16).

It is rare to detect dipterous fly larvae in human urine in Egypt; however, three cases of urinary myiasis with *Psychoda* spp. were reported from Egypt (5–7). A recent study had reported five cases with human urogenital myiasis caused by *Psychoda* spp. in Upper Egypt in Assuit and Qena Governorates (8). The first case report of human urinary myiasis due to larvae of *C. albipunctata* in Egypt was detected (3).

Ivermectin is semisynthetic macrocyclic lactone drug with antihelminthic activity. Nowadays, it is recommended as an important alternative for treatment of scabies, demodicidosis, head lice, and myiasis (17, 18).

In this case, we recommended the use of ivermectin as the clinical complaints continued for 2yr before diagnosis. The complaints regressed because of use of ivermectin in a single oral dose of 200ug/Kg, and increased fluid intake, without any recurrence of symptoms or larval passage for 2 months later. This result is in accordance with others who prescribed ivermectin in two oral doses of 200ug/kg (8). On the other hand, some clinicians had prescribed antibiotic and antiseptic treatment, accompanied with larval passage for 10d and then larvae passage and other patient symptoms disappeared (3).

This study reported and described the first case of human urinary myiasis caused by *C. albipunctata*in Beni-Suef Governorate, the second in Egypt and third case worldwide.

## Conclusion

Despite urinary myiasis is very rare in humans, urologists should consider it as an unusual cause of urinary tract infections especially in patients with non-specific urinary symptoms when no pathology is identified at routine examination and in those who live in rural areas with unsanitary poor environmental and socioeconomic levels.
